# Trajectories of frailty, grip strength and gait speed preceding dementia: a nested case–control study

**DOI:** 10.1093/ageing/afag062

**Published:** 2026-04-05

**Authors:** Zimu Wu, Aung Zaw Zaw Phyo, Lachlan Cribb, Swarna Vishwanath, Suzanne G Orchard, Alice Owen, Robyn Woods, Trevor T-J Chong, Rory Wolfe, Raj C Shah, Kerry Sheets, Anne Murray, Joanne Ryan

**Affiliations:** Monash University, School of Public Health and Preventive Medicine, Melbourne, Victoria, Australia; Monash University, School of Public Health and Preventive Medicine, Melbourne, Victoria, Australia; Monash University, School of Public Health and Preventive Medicine, Melbourne, Victoria, Australia; Monash University, School of Public Health and Preventive Medicine, Melbourne, Victoria, Australia; Monash University, School of Public Health and Preventive Medicine, Melbourne, Victoria, Australia; Monash University, School of Public Health and Preventive Medicine, Melbourne, Victoria, Australia; Monash University, School of Public Health and Preventive Medicine, Melbourne, Victoria, Australia; Monash University, Turner Institute for Brain and Mental Health, Clayton, Victoria, Australia; Alfred Health, Department of Neurology, Melbourne, Victoria, Australia; St Vincent's Hospital Melbourne Pty Ltd, Department of Clinical Neurosciences, Fitzroy, Victoria, Australia; Monash University, School of Public Health and Preventive Medicine, Melbourne, Victoria, Australia; Department of Family and Preventive Medicine and Rush Alzheimer’s Disease Center, Rush University Medical Center, Chicago, Illinois, USA; Geriatric Medicine Division, Department of Medicine, Hennepin Healthcare, Minneapolis, Minnesota, USA; Berman Center for Outcomes and Clinical Research, Hennepin Healthcare Research Institute, Minneapolis, Minnesota 55415, USA; Monash University, School of Public Health and Preventive Medicine, Melbourne, Victoria, Australia

**Keywords:** dementia, frailty, physical function, grip strength, gait speed, older people

## Abstract

**Background:**

Functional decline may be an early indicator of dementia. This study examined the trajectories of frailty, grip strength, and gait speed over the 11 years prior to dementia, compared to matched individuals without dementia.

**Methods:**

A total of 1092 dementia cases were matched on age, sex and education to 4368 controls from a cohort of community-dwelling older adults recruited in Australia and the USA, aged 65 years or above at recruitment. Frailty was characterised by a deficit-accumulation index involving 67 items. Hand grip strength and gait speed were measured regularly by physical examination. Linear mixed-effects models estimated the backward trajectories of frailty, grip strength and gait speed before dementia, compared to controls. Secondary analyses were stratified by sex and ApoE ε4 carrier status.

**Results:**

Higher frailty burden, with a steeper increase over time, was found in the years before dementia, compared to controls (*P*-interaction < .001). Hand grip strength and gait speed declined more rapidly in dementia cases than in controls (*P*-interaction < .001 for both). Differences between cases and controls became consistently significant four to six years prior to dementia (*P*-contrast < .001). An earlier divergence across all three measures was observed for females, and to a lesser extent in ApoE ε4 non-carriers.

**Discussion:**

Functional decline occurs within the decade before dementia onset, with gait speed being the earliest indicator. These findings support the utility of functional measures as early markers of dementia risk, with potential implications for targeted monitoring and preventative strategies.

## Key Points

- Frailty increased, while grip strength and gait speed declined over time. Frailty burden increased more rapidly in the years leading up to dementia.
- Grip strength and gait speed showed an accelerated decline before dementia.
- The earliest divergence was in gait speed, beginning ~6 years before dementia.
- Females showed earlier trajectory divergence than males across all three measures.

## Introduction

Dementia is a leading cause of disability and death worldwide, with its burden projected to increase over the next few decades due to an ageing population [1]. Although dementia is characterised by impairments in cognitive function, growing evidence suggests that deterioration in overall health, such as functional decline and increased multimorbidity, may occur years before dementia [2]. As such, measures that reflect broader aspects of health status may provide insights into the asymptomatic stages of dementia, which could support earlier intervention to delay health decline [3].

An important indicator of health status is frailty, which is broadly defined as a state of reduced physiological reserve and increased vulnerability associated with ageing [4, 5]. Frailty is typically measured in two ways: a continuous index quantified by the accumulation of a wide range of deficits (e.g. clinical conditions and diseases), and the Fried frailty phenotype, defined categorically based on the presence of pre-set clinical criteria, such as weakness and slowness [6–8]. Frailty has been increasingly recognised as a potential early marker of various adverse outcomes, including dementia and cognitive impairments [9, 10]. Moreover, as frailty develops progressively over time, investigating its trajectories using the frailty index, rather than relying on cross-sectional measurements, may offer a clearer picture of functional decline preceding dementia. One recent study observed an upward trend in frailty indices with an acceleration before dementia amongst individuals aged 60 years and above from combined cohorts in the USA and UK [11]. These results suggest that frailty, as an indicator of overall decline, may closely reflect brain ageing.

Hand grip strength and gait speed are widely used health indicators sensitive to many health outcomes [12, 13] and important tools used in frailty assessments [7, 14]. They are included in frailty indices if available, and are core components of the Fried Phenotype. Grip strength reflects the muscular strength of the upper limbs, while gait speed reflects the mobility, balance, and motor coordination of the lower limbs. Both measures have been prospectively associated with dementia risk and, more importantly, can be measured more easily than frailty [15]. As such, examining their trajectories may offer insights into the functional decline that occurs before dementia.

Using data from a cohort of community-dwelling older individuals aged 65 years or older, this study aimed to compare the retrospective trajectories of frailty, grip strength, and gait speed leading up to dementia and compare these trajectories to those of matched controls. Analyses were further stratified by sex and ApoE ε4 carrier status to determine whether trajectories vary according to these intrinsic biological factors, which modify risk of dementia and could alter physical functioning.

## Methods

### Study population

This study included participants from the ASPirin in Reducing Events in the Elderly (ASPREE) clinical trial and its observational follow-on study, ASPREE-eXTension. Details of these studies and participants have been reported elsewhere [16, 17]. In brief, participants were aged ≥65 years at study recruitment and were free from diagnosed dementia or major cognitive impairment (defined as Modified Mini-Mental State Examination (3MS) <78/100), cardiovascular disease, independence-limiting physical disability, or any illness that would be life-threatening within the first 5 years after study recruitment.

### Dementia ascertainment

Dementia was ascertained through a rigorous adjudication process, with details published previously [18]. Individuals with a suspected dementia diagnosis were first identified by the following criteria: a score of 77 or less on the 3MS, a decline of >10.15 points from the estimated score based on 3MS at study recruitment, adjusted by age and education, cognitive concerns self-reported or noted on medical records, a diagnosis of dementia by a clinician or prescription of cholinesterase inhibitors. Further evaluations were carried out at least six weeks after the initial dementia trigger, including the Alzheimer’s Disease Assessment Scale–Cognitive subscale, the Alzheimer's Disease Cooperative Study Activities of Daily Living scale, Colour Trails, and the Lurian overlapping figures. Additional materials, including laboratory results, brain imaging, and clinical case notes, were also collected. An international committee of geriatricians and neurologists, blinded to the study treatment arm, then assessed all available information and adjudicated dementia based on the Diagnostic and Statistical Manual of Mental Disorders, Fourth Edition (DSM-IV).

### Frailty assessment

Frailty was assessed annually over 11 years using a deficit-accumulation frailty index, comprising up to 67 items, developed following a standardised procedure for the ASPREE cohort. This index included a broad spectrum of health deficits, including 11 health conditions, 13 disease markers, 26 indicators of difficulties in daily activities, reduced quality of life, or physical limitations, 11 psychosocial and mental deficits, and 6 measures related to cognitive and physical function performance. The complete list of included deficits has been presented in Appendix 1, and detailed in our previous publication [14]. Each deficit was assigned a score ranging from 0 (absence) to 1 (presence). The frailty index score was calculated as the sum of deficits divided by the total number of items with available data. A frailty index score ranges from 0 to 1, with a higher score indicating a larger number of accumulated health deficits and thus greater frailty levels.

### Physical performance

Grip strength and gait speed were measured at study recruitment and repeatedly during follow-up [19]. Grip strength was measured in kilograms using a handheld isometric dynamometer (Jay-mar; JLW Instruments) on each hand. Participants were seated with their elbows flexed at 90 degrees during the test. Each participant completed three trials, at least 20 seconds apart, and the mean from the self-identified dominant hand was used for the analysis. Assessments were not conducted for those with hand injuries or pain. Walking time was assessed as the time taken in seconds to walk a 3-metre distance on a flat surface at a usual walking pace, from a standing start. At least 1 metre beyond the measured walking path was provided for deceleration. Two trials were conducted per participant. Gait speed was calculated as metres per second, with the average from the two trials used for the analysis.

### Matching

A nested case–control design was applied to select a subsample from the ASPREE cohort, as previously described [20]. Participants were excluded if they had missing data for frailty, grip strength and gait speed at study recruitment, or across all follow-up visits (*n* = 2295, 114 dementia cases). Comparison of baseline characteristics between participants included and excluded due to missing data is shown in Appendix 2. Amongst the remaining 16 819 participants, 1103 were diagnosed with dementia during the follow-up period. Each dementia case was matched to four controls at the study visit immediately preceding the time of dementia adjudication (the matching visit). Controls were selected from participants who were dementia-free and remained in follow-up at that matching visit (without replacement within and between visits). Matching was based on age at the matching visit (±1 year), sex (male, female), and years of education (11 or less, 12–15, 16 or more). In total, 1092 dementia cases were matched to 4368 controls, with 11 cases excluded due to insufficient matching (Appendix 3).

### Statistical analysis

Linear mixed-effects models with random intercepts and slopes for participants were used to estimate trajectories of frailty, grip strength, and gait speed. A backward timescale was used across a maximum of 12 study visits. Time 0 corresponds to the matching visit, which is up to ~1 year before the dementia trigger for cases, with a median lag time of 2.6 months (inter-quartile range: 0–9.9). The preceding years were coded sequentially from time −1 backwards, with inter-visit intervals ranging from 11.7 to 12.6 months. Models included case–control status, time terms (linear and quadratic), and their interactions, as well as age at time 0, sex, and education. Differences in trajectories between cases and controls were evaluated based on interactions between case–control status and time terms. A Wald test was performed to jointly assess the statistical significance of the two time terms (testing the null hypothesis that the trajectory in control participants was flat; *P*-values were referred to as *P*-change hereafter) and the two interaction terms (testing the null hypothesis that the shape of trajectories was the same between cases and controls, *P*-values were referred to as *P*-interaction hereafter). Mean differences between cases and controls at each time point were compared based on model-derived marginal means (*P*-values referred to as *P*-contrast hereafter).

Multiple sensitivity analyses were conducted to assess the robustness of the findings. First, the cognitive components were removed from the frailty index to characterise the non-cognitive frailty components in relation to dementia, given that cognitive decline occurs before dementia. Second, grip strength and gait speed were removed from the index to evaluate the extent to which the observed trajectories were independent of these physical-related factors. Third, we used spline-based models to explore whether alternative non-linear patterns existed beyond those captured by quadratic terms. Fourth, the missingness in ApoE genotype data (23.7% ASPREE participants) resulted in a reduced sample size and case–control imbalance in the original matched sample. Therefore, we repeated these analyses after performing matching separately within the ApoE ε4 carriers and non-carriers. Case–control matching was performed in Stata version 17.0 (StataCorp), and all subsequent analyses were conducted using R version 4.4.2. The ethical review committee of each participating university and institution approved both ASPREE and ASPREE-XT. All participants provided written informed consent.

## Results

Characteristics of the participants at study recruitment are shown in Table 1. The 1092 dementia cases had a mean age of 77.1 years at recruitment, and 45.7% were male. The 4368 controls had a similar demographic profile, with a mean age of 77.0 years at recruitment, and 45.7% were male. At time 0, the mean age was 82.2 years for cases and 82.1 years for controls. Compared to controls, cases were more likely to be ApoE ε4 carriers (*P* < .001), to have had diabetes (*P* = .007) or to have been frail (*P* < .001) at recruitment. However, they were less likely to be a current alcohol drinker (*P* = .02). Cases also had a higher death rate, fewer frailty, grip strength and gait speed assessments, and a shorter length of follow-up from recruitment compared to controls (Appendix 4).

**Table 1:** Participant characteristics at recruitment (*n* = 5460), by case–control status

	Cases (*n* = 1092)	Controls (*n* = 4368)	*P*-value^a^
	Mean (standard deviation)/no. (%)		
**Age at study recruitment, years**	77.1 (4.8)	77.0 (4.8)	.69
65–79	779 (71.3)	3160 (72.3)	.51
80+	313 (28.7)	1208 (27.7)	
**Age at matching visit, years**	82.2 (5.0)	82.1 (5.0)	.71
65–79	406 (37.2)	1620 (37.1)	.96
80+	686 (62.8)	2748 (62.9)	
**Sex**			1.00
Male	499 (45.7)	1996 (45.7)	
Female	593 (54.3)	2372 (54.3)	
**Years of education**			1.00
≤ 11	525 (48.1)	2100 (48.1)	
12–15	311 (28.5)	1244 (28.5)	
≥ 16	256 (23.4)	1024 (23.4)	
**ApoE ε4** ^b^			<.001
No	462 (56.0)	2689 (76.3)	
Yes	363 (44.0)	834 (23.7)	
**Smoking status**			.45
Never	638 (58.4)	2480 (56.8)	
Former	427 (39.1)	1756 (40.2)	
Current	27 (2.5)	132 (3.0)	
**Alcohol intake**			.02
Never	226 (20.7)	780 (17.9)	
Former	68 (6.2)	217 (5.0)	
Current	798 (73.1)	3371 (77.2)	
**Living situation**			.12
With someone or in residential/nursing homes	697 (63.8)	2896 (66.3)	
Living alone at home	395 (36.2)	1472 (33.7)	
**Hypertension** ^c^			.05
No	290 (26.6)	1038 (23.8)	
Yes	802 (73.4)	3330 (76.2)	
**Diabetes** ^d^			.007
No	953 (87.3)	3934 (90.1)	
Yes	139 (12.7)	434 (9.9)	
**Frailty** ^e^			<.001
Not frail	528 (48.4)	2486 (56.9)	
Pre-frail	521 (47.7)	1792 (41.0)	
Frail	43 (3.9)	90 (2.1)	

Abbreviation: ApoE, apolipoprotein E.

^a^The *P*-value is based on *t*-tests or chi-squared tests.

^b^There are 1112 missing values of ApoE ε4 carrier status in the case–control sample (*n* = 267 for cases, *n* = 845 for controls).

^c^Hypertension was defined as being on treatment for high blood pressure or blood pressure ≥140/90 mmHg at study recruitment.

^d^Diabetes was defined from self-report or as fasting glucose≥126 mg/dl or on treatment for diabetes.

^e^Frailty was defined based on the Fried frailty phenotype.

**Figure 1:**
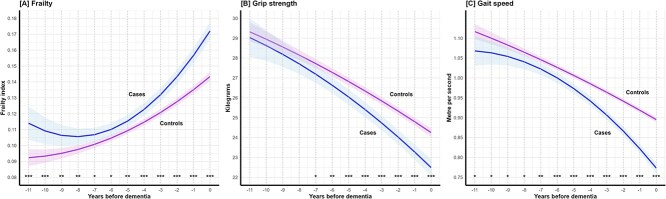
Mean trajectories of (A) frailty, (B) grip strength and (C) gait speed in cases preceding dementia (*n* = 1092) and in matched controls (*n* = 4368). Note: The solid lines and shadings represent the estimated mean trajectories and the 95% confidence intervals. ^*^P-contrast < .05; ^**^P-contrast < .01; ^***^P-contrast < .001.

Trajectories of frailty, grip strength and gait speed, including linear and quadratic terms, are presented relative to time 0, by case–control status in Figure 1 and Table 2. Increasing frailty was observed in both cases and controls (*P*-change < .001 for both; Appendix 5), but cases showed a greater increase over time (*P*-interaction < .001) and higher frailty levels throughout the follow-up than controls. Although differences in marginal means between cases and controls were shown across the follow-up, such differences became consistently significant from 4 years before dementia and became progressively larger approaching time 0 (*P*-contrast < .001 from times −4 to 0, Appendix 6).

**Table 2:** Differences in trajectories of frailty, grip strength and gait speed between dementia cases and matched controls (*n* = 5460)

	Overall (*n* = 5460)		Male (*n* = 2495)		Female (*n* = 2965)		ApoE ε4 carriers (*n* = 1197)^a^		ApoE ε4 non-carriers (n = 3151)^a^	
	Coefficients^b^ (95% CIs)	*P*-int^b^	Coefficients^b^ (95% CIs)	*P*-int^b^	Coefficients^b^ (95% CIs)	*P*-int^b^	Coefficients^b^ (95% CIs)	*P*-int^b^	Coefficients^b^ (95% CIs)	P-int^b^
**Frailty**										
**Time**	0.008 (0.006, 0.009)	<.001	0.009 (0.006, 0.011)	<.001	0.007 (0.005, 0.009)	<.001	0.004 (0.001, 0.007)	.001	0.012 (0.009, 0.014)	<0.001
**Time-squared**	0.001 (0.000, 0.001)		0.001 (0.000, 0.001)		0.001 (0.000, 0.001)		0.000 (−0.000, 0.001)		0.001 (0.001, 0.001)	
**Grip strength**										
**Time**	−0.249 (−0.397, −0.100)	<.001	−0.403 (−0.649, −0.157)	<.001	−0.133 (−0.307, 0.041)	.002	−0.142 (−0.419, 0.136)	.01	−0.350 (−0.557, −0.142)	<0.001
**Time-squared**	−0.010 (−0.028, 0.007)		−0.024 (−0.054, 0.006)		0.000 (−0.021, 0.021)		0.005 (−0.029, 0.039)		−0.026 (−0.051, −0.002)	
**Gait speed**										
**Time**	−0.027 (−0.033, −0.021)	<.001	−0.028 (−0.037, −0.020)	<.001	−0.026 (−0.034, −0.018)	<.001	−0.029 (−0.040, −0.017)	<.001	−0.025 (−0.033, −0.016)	<0.001
**Time-squared**	−0.002 (−0.003, −0.001)		−0.002 (−0.003, −0.001)		−0.002 (−0.003, −0.001)		−0.002 (−0.003, −0.001)		−0.002 (−0.003, −0.001)	

Abbreviations: ApoE, apolipoprotein E; *P*-int, *P*-interaction.

^a^Analysis stratified by ApoE ε4 carrier status included 4348 participants, with 1197 carriers (363 cases and 834 controls) and 3151 non-carriers (462 cases and 2689 controls).

^b^The coefficients and *P*-interaction represent the estimated differences in linear and quadratic time effects between cases and controls (reference group). Models included case–control status, time, time-squared, and their interactions, as well as age at time 0, sex, and years of education. The *P*-interaction was derived from a joint test of the interaction terms. A significant *P*-interaction suggests a difference in the trajectories of change between cases and controls (in either the linear or quadratic component, or both).

A decline in grip strength was also shown in both cases and controls (*P*-change < .001 for both; Figure 1, Appendix 5). However, cases declined faster compared to controls (*P*-interaction < .001) and had significantly lower grip strength than controls at each time point from 5 years before dementia (*P*-contrast < .001 from times −5 to 0, Appendix 7). Gait speed also declined in both subgroups (*P*-change < .001 for both; Appendix 5). Similar to grip strength, cases showed a steeper decline compared to controls (*P*-interaction < .001), as well as significantly slower gait speed as early as 6 years before diagnosis (*P*-contrast < .001 from time −6 to 0, Appendix 8).

**Figure 2:**
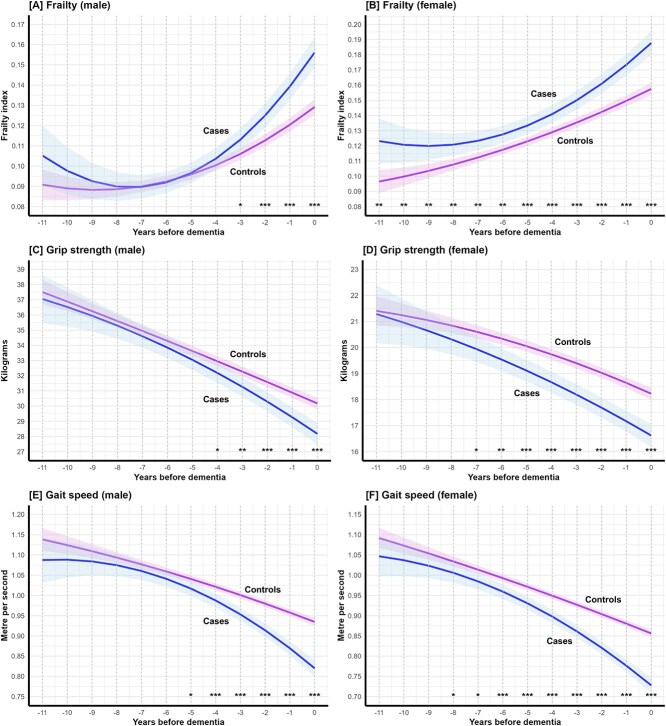
Mean trajectories of (A, B) frailty, (C, D) grip strength, (E, F) gait speed in cases preceding dementia and in matched controls amongst males (499 cases and 1996 controls) and females (593 cases and 2372 controls). Note: The solid lines and shadings represent the estimated mean trajectories and the 95% confidence intervals. ^*^P-contrast < .05; ^**^P-contrast < .01; ^***^P-contrast < .001.

**Figure 3:**
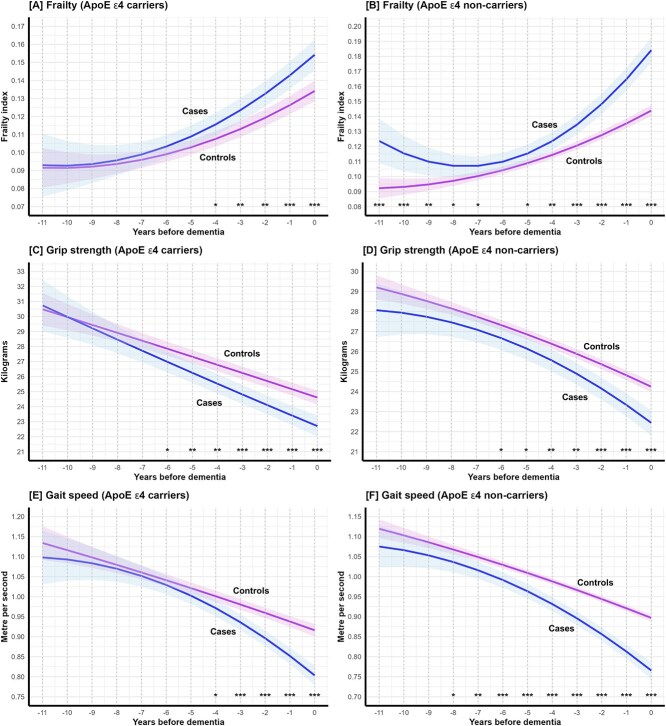
Mean trajectories of (A, B) frailty, (C, D) grip strength, (E, F) gait speed in cases preceding dementia and in matched controls amongst ApoE ε4 carriers (363 cases and 834 controls) and non-carriers (462 cases and 2689 controls). Note: The solid lines and shadings represent the estimated mean trajectories and the 95% confidence intervals. ^*^P-contrast < .05; ^**^P-contrast < .01; ^***^P-contrast < .001.

In general, subgroup analyses were consistent with the main analyses, but the timing of trajectory divergence between cases and controls varied by subgroup. Sex-stratified analyses (Figure 2, Table 2) showed that significant divergence in frailty, grip strength, and gait speed (*P*-contrast < .001) occurred earlier in females than in males (Appendix 9–14). When stratified by ApoE ε4 status, the timing of divergence in frailty and grip strength was generally similar between carriers and non-carriers (Figure 3, Table 2, Appendix 15–18). However, non-carriers showed earlier divergence for gait speed (*P*-contrast < .001, Appendix 19 and 20), although their sample size was larger than that of carriers (*n* = 3151 versus 1197).

Trajectories derived from unadjusted models showed similar patterns and are presented in Appendix 21. The sensitivity analyses did not show any result that was materially different from those of the main analyses (Appendix 22–25).

## Discussion

In this nested case–control analysis, we present evidence of functional decline in the years preceding a clinical diagnosis of dementia. An earlier and steeper deterioration was shown in frailty, grip strength and gait speed amongst individuals who eventually developed dementia compared to those who did not. A greater burden of frailty with a faster increase over time was observed in dementia cases than in controls without dementia. Likewise, grip strength and gait speed both declined more rapidly in cases. Notably, significant differences between cases and controls became consistently evident starting from 6 years for gait speed, 5 years for hand grip strength, and 4 years for frailty before dementia. The trajectory divergence of all measures generally appeared earlier in females than in males. The current findings indicate that clear functional decline may begin 4–6 years before dementia, with routine physical assessments, particularly gait speed, standing out as feasible and sensitive early markers.

Our findings support the growing evidence that frailty is a risk factor for cognitive impairment, with a strong association with incident dementia [21, 22]. Whether assessed via the Fried phenotype or deficit accumulation index [5, 7, 8], these two approaches are increasingly viewed as complementary, capturing different dimensions of frailty. As previous studies have largely focused on the Fried phenotype, our study adds value by examining both a comprehensive frailty index and two core components of the Fried phenotype separately. Moreover, we found that these components alone showed trajectories of decline that were similarly steep, and in some cases diverged even earlier than those of the frailty index in the years leading up to dementia. This raises the important question of whether simpler physical assessments may be sufficient for early identification of dementia risk. However, as noted in previous studies [23, 24], a significant advantage of the frailty index is its capacity for automated derivation from routinely collected clinical data and diagnostic codes within electronic health records. This enables frailty assessment to be integrated into existing clinical workflows, particularly in primary care settings, without the need for additional time-consuming in-person testing.

To our knowledge, evidence regarding the longitudinal patterns of frailty preceding dementia remains limited. One recent multi-cohort study examined frailty trajectories across four cohorts of older individuals aged above 60 years [11]. The authors observed an acceleration in the frailty index 4–9 years before the onset of dementia. Our findings closely align with these previous observations. Specifically, the divergence in frailty between cases and controls became consistently significant, starting around 4 years before dementia. When cognitive components were removed from the index (an approach similar to that employed previously [11]), the divergence shifted slightly later but remained consistent with the previously reported longitudinal patterns. This indicates that while earlier frailty differences between cases and controls may be partly driven by subtle cognitive changes, non-cognitive deterioration also provides a clear prodromal signal. Complementing the evidence from that larger combined cohort, our study provides an important validation in an entirely separate population, further reinforcing the generalisability of these prodromal patterns.

Grip strength and gait speed are important components of geriatric assessment, widely recognised as markers for physical capacity and overall health in older adults [25, 26]. Apart from their associations with disability and mortality, accumulating evidence has also linked both measures to cognitive impairments and dementia [12, 27–30]. Lower levels of muscle strength and mobility are believed to reflect not only age-related declines in the musculoskeletal system but also neuropathology, such as cerebrovascular damage, brain atrophy and amyloid accumulation [25, 31, 32]. A number of previous studies have shown associations of both measures with an increased risk of cognitive decline and dementia [15, 27, 28, 33]. Specifically, previous findings from this cohort showed that grip strength and gait speed are complementary in dementia risk assessment, with their combination providing a stronger prediction [15]. However, their changing patterns during the years before dementia could still be better understood. Of note, gait speed declined earlier than grip strength in our study, suggesting that walking ability may be more sensitive to brain changes than upper limb strength. One possible explanation is that walking is a complex coordinative activity associated with a wide range of health factors, such as musculoskeletal conditions, motor control, sensor function, and social interaction [34, 35]. This may be more affected by neurodegeneration compared to the mass, strength, and function of peripheral muscle, reflected by grip strength [36, 37].

One interesting finding is that females showed an earlier divergence of frailty trajectories from controls than males, with greater frailty burden across the follow-up. This complements prior literature showing that females generally have higher frailty levels and disability rates than males across the life course [38, 39]. While the reasons for this finding remain uncertain, it is plausible that lower functional capacity and physiological reserve in females may make even slight functional decline more observable. The earlier divergence observed in ApoE ε4 non-carriers may be attributed to different pathological pathways to dementia. Alzheimer’s disease is thought to be primarily driven by amyloid-related changes in ApoE ε4 carriers, whereas in non-carriers, the pathological mechanisms may be more heterogeneous, involving broader physiological systems [40, 41].

One strength of the current study is the use of a novel multidimensional frailty index, which integrates a wide range of health deficits across physical, biological and clinical domains. The index has shown excellent construct, concurrent and predictive validity across demographic characteristics and key health outcomes of older individuals [14]. This ensures a solid and comprehensive measure of health deficits. Another strength is that the items of the frailty index, as well as grip strength and gait speed, were assessed by trained staff according to a pre-defined protocol [19]. Disease diagnoses in the frailty index and incident dementia [18] were all adjudicated by international expert panels of clinicians, in reference to a variety of medical evidence to ensure diagnostic accuracy. This minimises the risk of measurement error or misclassification. Moreover, the case–control design strengthened comparability between the two subgroups in key sociodemographic aspects. Longitudinal modelling with time-specific comparison between cases and controls provides detailed information for the patterns of functional changes over time. Meanwhile, limitations need to be acknowledged. The study sample was drawn from the ASPREE participants, who were older individuals without major cognitive impairments, independence-limiting physical disability, or known 5-year life-threatening diseases. Furthermore, participants excluded from our analysis due to missing data had worse baseline health status compared to those included, suggesting that our sample may represent a healthier subset of the already-healthy ASPREE cohort. Another limitation is that, because controls were matched to resemble cases to improve comparability, their trajectories may not fully reflect those of the wider dementia-free population. These may potentially limit the generalisability of our findings. Moreover, the quadratic model may not fully capture the trajectory shape. Although alternative non-linear forms could be explored, we did not select them based on observed patterns to avoid potential overfitting.

## Supplementary Material

aa-25-3172-File005_afag062

## References

[1] Collaborators GBDDF . Estimation of the global prevalence of dementia in 2019 and forecasted prevalence in 2050: an analysis for the global burden of disease study 2019. Lancet Public Health 2022;7:e105–25. 10.1016/S2468-2667(21)00249-8. 34998485 PMC8810394

[2] You J, Guo Y, Wang YJ et al. Clinical trajectories preceding incident dementia up to 15 years before diagnosis: a large prospective cohort study. Mol Psychiatry 2024;29:3097–105. 10.1038/s41380-024-02570-0. 38678085

[3] Rasmussen J, Langerman H. Alzheimer's disease - why we need early diagnosis. Degener Neurol Neuromuscul Dis 2019;9:123–30. 10.2147/DNND.S228939. 31920420 PMC6935598

[4] Doody P, Lord JM, Greig CA et al. Frailty: pathophysiology, theoretical and operational definition(s), impact, prevalence, management and prevention, in an increasingly economically developed and ageing world. Gerontology. 2023;69:927–45. 10.1159/000528561. 36476630 PMC10568610

[5] Kim DH, Rockwood K. Frailty in older adults. N Engl J Med 2024;391:538–48. 10.1056/NEJMra2301292. 39115063 PMC11634188

[6] Rockwood K, Mitnitski A. Frailty in relation to the accumulation of deficits. J Gerontol A Biol Sci Med Sci 2007;62:722–7. 10.1093/gerona/62.7.722. 17634318

[7] Fried LP, Tangen CM, Walston J et al. Frailty in older adults: evidence for a phenotype. J Gerontol A Biol Sci Med Sci 2001;56:M146–57. 10.1093/gerona/56.3.m146. 11253156

[8] Cesari M, Gambassi G, van Kan GA et al. The frailty phenotype and the frailty index: different instruments for different purposes. Age Ageing 2014;43:10–2. 10.1093/ageing/aft160. 24132852

[9] Vermeiren S, Vella-Azzopardi R, Beckwee D et al. Frailty and the prediction of negative health outcomes: a meta-analysis. J Am Med Dir Assoc 2016;17:1163.e1. 10.1016/j.jamda.2016.09.010. 27886869

[10] Livingston G, Huntley J, Liu KY et al. Dementia prevention, intervention, and care: 2024 report of the lancet standing commission. Lancet. 2024;404:572–628. 10.1016/S0140-6736(24)01296-0. 39096926

[11] Ward DD, Flint JP, Littlejohns TJ et al. Frailty trajectories preceding dementia in the US and UK. JAMA Neurol 2025;82:61–71. 10.1001/jamaneurol.2024.3774. 39527039 PMC11555573

[12] Soysal P, Hurst C, Demurtas J et al. Handgrip strength and health outcomes: umbrella review of systematic reviews with meta-analyses of observational studies. J Sport Health Sci 2021;10:290–5. 10.1016/j.jshs.2020.06.009. 32565244 PMC8167328

[13] Abellan van Kan G, Rolland Y, Andrieu S et al. Gait speed at usual pace as a predictor of adverse outcomes in community-dwelling older people an international academy on nutrition and aging (IANA) task force. J Nutr Health Aging 2009;13:881–9. 10.1007/s12603-009-0246-z. 19924348 PMC12878092

[14] Ryan J, Espinoza S, Ernst ME et al. Validation of a deficit-accumulation frailty index in the ASPirin in reducing events in the elderly study and its predictive capacity for disability-free survival. J Gerontol A Biol Sci Med Sci 2022;77:19–26. 10.1093/gerona/glab225. 34338761 PMC8751791

[15] Orchard SG, Polekhina G, Ryan J et al. Combination of gait speed and grip strength to predict cognitive decline and dementia. Alzheimers Dement (Amst) 2022;14:e12353. 10.1002/dad2.12353. 36187193 PMC9494608

[16] Ernst ME, Broder JC, Wolfe R et al. Health characteristics and aspirin use in participants at the baseline of the ASPirin in reducing events in the elderly - eXTension (ASPREE-XT) observational study. Contemp Clin Trials 2023;130:107231. 10.1016/j.cct.2023.107231. 37196887 PMC10330669

[17] McNeil JJ, Woods RL, Nelson MR et al. Baseline characteristics of participants in the ASPREE (ASPirin in reducing events in the elderly) study. J Gerontol A Biol Sci Med Sci 2017;72:1586–93. 10.1093/gerona/glw342. 28329340 PMC5861878

[18] Ryan J, Storey E, Murray AM et al. Randomized placebo-controlled trial of the effects of aspirin on dementia and cognitive decline. Neurology. 2020;95:e320–31. 10.1212/WNL.0000000000009277. 32213642 PMC7455352

[19] Wolfe R, Murray AM, Woods RL et al. The aspirin in reducing events in the elderly trial: statistical analysis plan. Int J Stroke 2018;13:335–8. 10.1177/1747493017741383. 29111960 PMC6380180

[20] Wu Z, Cribb L, Wolfe R et al. Cardiometabolic trajectories preceding dementia in community-dwelling older individuals. JAMA Netw Open 2025;8:e2458591. 10.1001/jamanetworkopen.2024.58591. 39918818 PMC11806394

[21] Kojima G, Taniguchi Y, Iliffe S et al. Frailty as a predictor of Alzheimer disease, vascular dementia, and all dementia among community-dwelling older people: a systematic review and meta-analysis. J Am Med Dir Assoc 2016;17:881–8. 10.1016/j.jamda.2016.05.013. 27324809

[22] Petermann-Rocha F, Lyall DM, Gray SR et al. Associations between physical frailty and dementia incidence: a prospective study from UK biobank. Lancet Healthy Longev 2020;1:e58–68. 10.1016/S2666-7568(20)30007-6. 36094146

[23] Brack C, Kynn M, Murchie P et al. Validated frailty measures using electronic primary care records: a review of diagnostic test accuracy. Age Ageing 2023;52:1–8. 10.1093/ageing/afad173. PMC10873280 37993406

[24] Kochar B, Cheng D, Lehto HR et al. Application of an electronic frailty index to identify high-risk older adults using electronic health record data. J Am Geriatr Soc 2025;73:1491–7. 10.1111/jgs.19389. 39982448 PMC12101934

[25] Bohannon RW . Grip strength: An indispensable biomarker for older adults. Clin Interv Aging 2019;14:1681–91. 10.2147/CIA.S194543. 31631989 PMC6778477

[26] Peel NM, Kuys SS, Klein K. Gait speed as a measure in geriatric assessment in clinical settings: a systematic review. J Gerontol A Biol Sci Med Sci 2013;68:39–46. 10.1093/gerona/gls174. 22923430

[27] Zammit AR, Robitaille A, Piccinin AM et al. Associations between aging-related changes in grip strength and cognitive function in older adults: a systematic review. J Gerontol A Biol Sci Med Sci 2019;74:519–27. 10.1093/gerona/gly046. 29528368 PMC6417444

[28] Peel NM, Alapatt LJ, Jones LV et al. The association between gait speed and cognitive status in community-dwelling older people: a systematic review and meta-analysis. J Gerontol A Biol Sci Med Sci 2019;74:943–8. 10.1093/gerona/gly140. 29917045

[29] Cesari M . Role of gait speed in the assessment of older patients. JAMA. 2011;305:93–4. 10.1001/jama.2010.1970. 21205972

[30] Xie X, Li D, Zhou M et al. Effects of hand strength and walking speed combined and in isolation on the prediction of cognitive decline and dementia in middle-aged and older adults: a systematic review and meta-analysis. J Am Med Dir Assoc 2025;26:105576. 10.1016/j.jamda.2025.105576. 40157391

[31] Demnitz N, Zsoldos E, Mahmood A et al. Associations between mobility, cognition, and brain structure in healthy older adults. Front Aging Neurosci 2017;9:155. 10.3389/fnagi.2017.00155. 28588477 PMC5440513

[32] Skillback T, Blennow K, Zetterberg H et al. Slowing gait speed precedes cognitive decline by several years. Alzheimers Dement 2022;18:1667–76. 10.1002/alz.12537. 35142034 PMC9514316

[33] Wu Z, Woods RL, Chong TT et al. Grip strength, gait speed, and trajectories of cognitive function in community-dwelling older adults: a prospective study. Alzheimers Dement (Amst) 2023;15:e12388. 10.1002/dad2.12388. 36815873 PMC9927855

[34] Fritz S, Lusardi M. White paper: ``walking speed: the sixth vital sign''. J Geriatr Phys Ther 2009;32:2–5. 10.1519/00139143-200932020-00002. 20039582

[35] Shankar A, McMunn A, Demakakos P et al. Social isolation and loneliness: prospective associations with functional status in older adults. Health Psychol 2017;36:179–87. 10.1037/hea0000437. 27786518

[36] Ambike S, Paclet F, Zatsiorsky VM et al. Factors affecting grip force: anatomy, mechanics, and referent configurations. Exp Brain Res 2014;232:1219–31. 10.1007/s00221-014-3838-8. 24477762 PMC4013148

[37] Vaishya R, Misra A, Vaish A et al. Hand grip strength as a proposed new vital sign of health: a narrative review of evidences. J Health Popul Nutr 2024;43:7. 10.1186/s41043-024-00500-y. 38195493 PMC10777545

[38] Gordon EH, Peel NM, Samanta M et al. Sex differences in frailty: a systematic review and meta-analysis. Exp Gerontol 2017;89:30–40. 10.1016/j.exger.2016.12.021. 28043934

[39] Park C, Ko FC. The science of frailty: sex differences. Clin Geriatr Med 2021;37:625–38. 10.1016/j.cger.2021.05.008. 34600727 PMC8493788

[40] Jiang S, Tang L, Zhao N et al. A systems view of the differences between APOE epsilon4 carriers and non-carriers in Alzheimer's disease. Front Aging Neurosci 2016;8:171. 10.3389/fnagi.2016.00171. 27462267 PMC4941795

[41] Monsell SE, Kukull WA, Roher AE et al. Characterizing Apolipoprotein E epsilon4 carriers and noncarriers with the clinical diagnosis of mild to moderate Alzheimer dementia and minimal beta-amyloid peptide plaques. JAMA Neurol 2015;72:1124–31. 10.1001/jamaneurol.2015.1721. 26302353 PMC4833059

