# Supplementary material for: Trajectories of frailty, grip strength and gait speed preceding dementia: a nested case–control study
Source: Age Ageing. 2026 Apr 5;55(4):afag062. doi: 10.1093/ageing/afag062 (PMC13070003; doi:10.1093/ageing/afag062)
Supplement: aa-25-3172-File005_afag062 [file aa-25-3172-file005_afag062.docx]

**Appendix 1. Items of the ASPREE frailty index**

| **Disease diagnosis (11)** |
| --- |
| Cancer, depression, diabetes, stroke, major haemorrhage, myocardial infarction, gastroesophageal reflux, hospitalisation for heart failure, osteoarthritis, osteoporosis, urinary incontinence |
| **Disease indicators (13)** |
| Albuminuria, anaemia, central adiposity, dyslipidaemia, hospitalisations in the last year, hypertension, hypotension, number of drugs, obesity, chronic kidney disease, self-rated health status, smoking, underweight |
| **Functional decline (26)** |
| Difficulty with bathing, difficulty with dressing, difficulty with eating, difficulty with toileting, difficulty with transferring, difficulty walking, accomplished less, climbing stairs limited, limited in kind of work/activities, moderate activities limited, pain interfere with work, requires a walking aid, any walking outside, difficulty walking several blocks, difficulty walking 8-12 blocks, difficulty climbing 1 flight of stairs, difficulty getting into a car, difficulty gripping objects, difficulty lifting 10 pounds, difficulty lifting heavy objects, difficulty raising arms above head, difficulty running errands, difficulty walking 1 block, difficulty with light housework, difficulty with meal preparation, difficulty with shopping |
| **Mental & Psychosocial (11)** |
| Accomplished less due to emotional problems, a lot of energy, calm or peaceful, difficulty participating in community activities, difficulty taking care of family, difficulty using the telephone, difficulty with financial management, difficulty visiting relatives/friends, downhearted and depressed, less careful in work/activities, social activities impacted by health |
| **Cognition & Performance (6)** |
| episodic memory, low global cognition, low psychomotor speed, low language and executive function, slow gait speed over 3 metres, weak handgrip strength |

Note: For further details on items, sources and scoring & cut-offs, please refer to: <https://doi.org/10.1093/gerona/glab225>.

**Appendix 2. Comparison of participants included and excluded from matching due to missing data (n=19, 114)**

|  | **Excluded (n=2, 295)** | **Included (n=16, 819)** | **P-value ^a^** |
| --- | --- | --- | --- |
|  | **Mean (standard deviation) / No. (%)** | | |
|  |  |  |  |
| **Age at study recruitment, years** | 75.92 (5.29) | 75.00 (4.42) | <0.001 |
| 65-79 | 1, 781 (77.6) | 14, 405 (85.6) | <0.001 |
| 80+ | 514 (22.4) | 2, 414 (14.4) |  |
| **Sex** |  |  | <0.001 |
| Male | 916 (39.9) | 7, 416 (44.1) |  |
| Female | 1, 379 (60.1) | 9, 403 (55.9) |  |
| **Years of education** |  |  | <0.001 |
| ≤11 | 1, 110 (48.4) | 7, 526 (44.8) |  |
| 12-15 | 706 (30.8) | 4, 868 (28.9) |  |
| ≥16 | 478 (20.8) | 4, 425 (26.3) |  |
| **ApoE ε4** |  |  | 0.23 |
| No | 777 (72.7) | 10, 040 (74.3) |  |
| Yes | 292 (27.3) | 3, 466 (25.7) |  |
| **Smoking status** |  |  | <0.001 |
| Never | 1, 177 (51.3) | 9, 403 (55.9) |  |
| Former | 950 (41.4) | 6, 849 (40.7) |  |
| Current | 168 (7.3) | 567 (3.4) |  |
| **Alcohol intake** |  |  | <0.001 |
| Never | 476 (20.7) | 2, 860 (17.0) |  |
| Former | 206 (9.0) | 930 (5.5) |  |
| Current | 1, 613 (70.3) | 13, 029 (77.5) |  |
| **Living situation** |  |  | <0.001 |
| With someone or in residential/nursing homes | 1, 402 (61.1) | 11, 461 (68.1) |  |
| Living alone at home | 893 (38.9) | 5, 358 (31.9) |  |
| **Hypertension** |  |  | 0.07 |
| No | 555 (24.2) | 4, 363 (25.9) |  |
| Yes | 1, 740 (75.8) | 12, 456 (74.1) |  |
| **Diabetes** |  |  | <0.001 |
| No | 1, 999 (87.1) | 15, 070 (89.6) |  |
| Yes | 296 (12.9) | 1, 749 (10.4) |  |
| **Frailty** |  |  | <0.001 |
| Not frail | 1, 109 (48.3) | 10, 136 (60.3) |  |
| Pre-frail | 1, 088 (47.4) | 6, 359 (37.8) |  |
| Frail | 98 (4.3) | 324 (1.9) |  |

^a^ P-values were derived from t-tests or chi-squared tests.

**
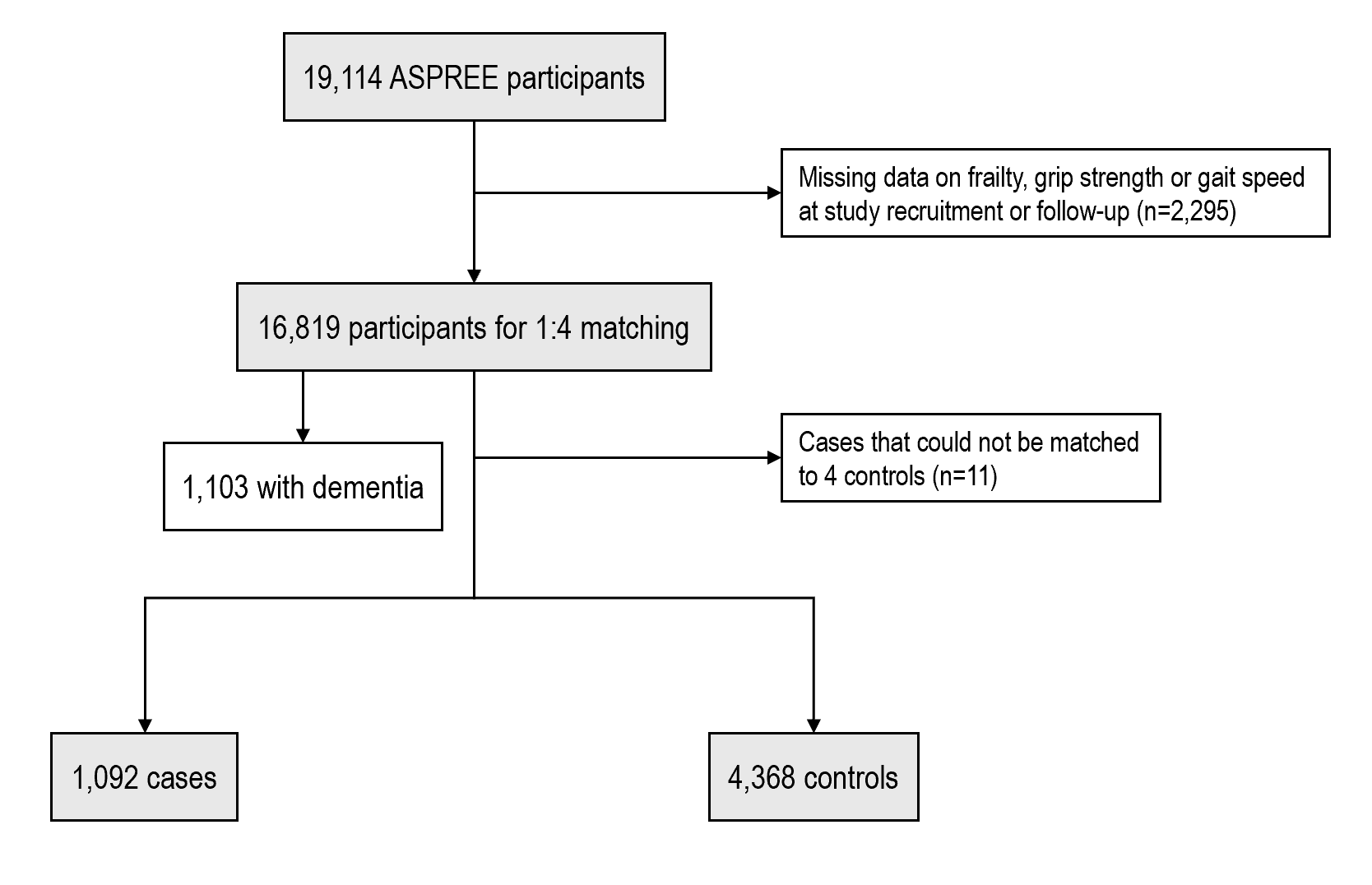
**

**Appendix 3. Flowchart of participant selection**

**Appendix 4. Data availability by case-control status (n=5, 460)**

|  | **Cases (n=1, 092)** | **Controls (n=4, 368)** | **P-value ^a^** |
| --- | --- | --- | --- |
| **Death (n, %)** | 318 (29.1) | 534 (12.2) | <0.001 |
| **Number of study visits with available measures (median, IQR)** |  |  |  |
| **Frailty** | 8 (6-9) | 9 (8-10) | <0.001 |
| **Grip strength** | 4 (3-6) | 5 (4-6) | <0.001 |
| **Gait speed** | 4 (3-6) | 5 (4-6) | <0.001 |
| **Years of follow-up (median, IQR) ^b^** | 8.26 (6.53-9.53) | 9.22 (7.96-10.27) | <0.001 |

^a^ P-values were derived from chi-squared tests or Wilcoxon rank-sum tests.

^b^ Years of follow-up were defined as the time from study recruitment to the last secondary outcome screening.

**Appendix 5. Time coefficients of trajectories for dementia cases and matched controls (n=5, 460)**

|  | **Cases (n=1, 092)** | | | **Controls (n=4, 368)** | | |
| --- | --- | --- | --- | --- | --- | --- |
|  | **Time ^a^** | **Time-squared ^a^** | **P-change ^b^** | **Time ^a^** | **Time-squared ^a^** | **P-change ^b^** |
| **Overall** |  |  |  |  |  |  |
| **Frailty** | 0.016 (0.015, 0.018) | 0.001 (0.001, 0.001) | <0.001 | 0.009 (0.006, 0.009) | 0.000 (0.000, 0.001) | <0.001 |
| **Grip strength** | -0.806 (-0.939, -0.674) | -0.019 (-0.035, -0.003) | <0.001 | -0.558 (-0.624, -0.491) | -0.009 (-0.017, -0.001) | <0.001 |
| **Gait speed** | -0.051 (-0.056, -0.046) | -0.002 (-0.003, -0.002) | <0.001 | -0.024 (-0.027, -0.020) | -0.000 (-0.001, -0.000) | <0.001 |
| **Males** |  |  |  |  |  |  |
| **Frailty** | 0.018 (0.016, 0.020) | 0.001 (0.001, 0.001) | <0.001 | 0.009 (0.008, 0.010) | 0.001 (0.000, 0.001) | <0.001 |
| **Grip strength** | -1.112 (-1.333, -0.892) | -0.028 (-0.054, -0.001) | <0.001 | -0.709 (-0.818, -0.600) | -0.004 (-0.017, 0.009) | <0.001 |
| **Gait speed** | -0.052 (-0.060, -0.044) | -0.003 (-0.003, -0.002) | <0.001 | -0.023 (-0.027, -0.020) | -0.000 (-0.001, 0.000) | <0.001 |
| **Females** |  |  |  |  |  |  |
| **Frailty** | 0.015 (0.013, 0.017) | 0.001 (0.001, 0.001) | <0.001 | 0.008 (0.007, 0.010) | 0.000 (0.000, 0.001) | <0.001 |
| **Grip strength** | -0.562 (-0.717, -0.408) | -0.013 (-0.031, 0.006) | <0.001 | -0.429 (-0.509, -0.350) | -0.013 (-0.022, -0.003) | <0.001 |
| **Gait speed** | -0.050 (-0.057, -0.043) | -0.002 (-0.003, -0.001) | <0.001 | -0.024 (-0.028, -0.021) | -0.000 (-0.001, 0.000) | <0.001 |
| **ApoE ε4 carriers** |  |  |  |  |  |  |
| **Frailty** | 0.012 (0.010, 0.014) | 0.001 (0.000, 0.001) | <0.001 | 0.008 (0.007, 0.010) | 0.000 (0.000, 0.001) | <0.001 |
| **Grip strength** | -0.691 (-0.922, -0.459) | 0.004 (-0.025, 0.032) | <0.001 | -0.549 (-0.702, -0.396) | -0.001 (-0.020, 0.017) | <0.001 |
| **Gait speed** | -0.051 (-0.060, -0.041) | -0.002 (-0.003, -0.001) | <0.001 | -0.022 (-0.028, -0.016) | -0.000 (-0.001, 0.001) | <0.001 |
| **ApoE ε4 non-carriers** |  |  |  |  |  |  |
| **Frailty** | 0.021 (0.019, 0.023) | 0.001 (0.001, 0.002) | <0.001 | 0.009 (0.008, 0.010) | 0.000 (0.000, 0.000) | <0.001 |
| **Grip strength** | -0.936 (-1.126, -0.745) | -0.039 (-0.061, -0.016) | <0.001 | -0.586 (-0.668, -0.503) | -0.012 (-0.022, -0.002) | <0.001 |
| **Gait speed** | -0.049 (-0.057, -0.041) | -0.002 (-0.003, -0.001) | <0.001 | -0.024 (-0.028, -0.021) | -0.000 (-0.001, 0.000) | <0.001 |

^a^ The coefficients represent the estimated trajectories of change over time within cases and controls. Models included case-control status, time, time-squared, and their interactions, as well as age at time 0, sex, and years of education.

^b^ The p-change was derived from a joint test of time and time-squared. A significant p-change suggests time effects in either the linear or quadratic component, or both.

**Appendix 6. Estimated marginal means of frailty by case-control status (n=5,460)**

|  | **Estimated marginal means (95% CIs)** | |  |  |
| --- | --- | --- | --- | --- |
| **Time** | **Cases** | **Controls** | **P-contrast ^a^** | **Adjusted p-contrast ^b^** |
| **-11** | 0.114 (0.104, 0.124) | 0.092 (0.087, 0.098) | <0.001 | <0.001 |
| **-10** | 0.109 (0.101, 0.117) | 0.093 (0.089, 0.098) | <0.001 | 0.002 |
| **-9** | 0.106 (0.100, 0.113) | 0.095 (0.092, 0.098) | 0.002 | 0.003 |
| **-8** | 0.106 (0.101, 0.111) | 0.098 (0.095, 0.100) | 0.005 | 0.006 |
| **-7** | 0.107 (0.103, 0.111) | 0.101 (0.099, 0.103) | 0.01 | 0.01 |
| **-6** | 0.110 (0.106, 0.114) | 0.105 (0.103, 0.107) | 0.01 | 0.01 |
| **-5** | 0.115 (0.112, 0.119) | 0.109 (0.107, 0.111) | 0.005 | 0.006 |
| **-4** | 0.123 (0.119, 0.127) | 0.115 (0.113, 0.117) | <0.001 | <0.001 |
| **-3** | 0.132 (0.128, 0.136) | 0.121 (0.119, 0.123) | <0.001 | <0.001 |
| **-2** | 0.143 (0.139, 0.148) | 0.128 (0.125, 0.130) | <0.001 | <0.001 |
| **-1** | 0.157 (0.152, 0.161) | 0.135 (0.133, 0.138) | <0.001 | <0.001 |
| **0** | 0.172 (0.167, 0.177) | 0.143 (0.141, 0.146) | <0.001 | <0.001 |

^a^ P-values for comparisons between cases and controls at each specific time point.

^b^ P-values adjusted using the False Discovery Rate (FDR) procedure.

**Appendix 7. Estimated marginal means of grip strength by case-control status (n=5,460)**

|  | **Estimated marginal means (95% CIs)** | |  |  |
| --- | --- | --- | --- | --- |
| **Time** | **Cases** | **Controls** | **P-contrast ^a^** | **Adjusted p-contrast ^b^** |
| **-11** | 29.031 (28.066, 29.995) | 29.322 (28.837, 29.806) | 0.60 | 0.60 |
| **-10** | 28.628 (27.857, 29.398) | 28.948 (28.560, 29.336) | 0.47 | 0.51 |
| **-9** | 28.187 (27.569, 28.804) | 28.557 (28.245, 28.869) | 0.29 | 0.35 |
| **-8** | 27.707 (27.201, 28.213) | 28.148 (27.891, 28.405) | 0.12 | 0.17 |
| **-7** | 27.189 (26.753, 27.624) | 27.722 (27.500, 27.945) | 0.03 | 0.05 |
| **-6** | 26.632 (26.235, 27.030) | 27.278 (27.074, 27.482) | 0.004 | 0.007 |
| **-5** | 26.037 (25.657, 26.417) | 26.817 (26.622, 27.012) | <0.001 | <0.001 |
| **-4** | 25.404 (25.033, 25.774) | 26.338 (26.147, 26.529) | <0.001 | <0.001 |
| **-3** | 24.732 (24.369, 25.095) | 25.842 (25.655, 26.029) | <0.001 | <0.001 |
| **-2** | 24.022 (23.662, 24.381) | 25.328 (25.142, 25.514) | <0.001 | <0.001 |
| **-1** | 23.273 (22.902, 23.643) | 24.797 (24.605, 24.988) | <0.001 | <0.001 |
| **0** | 22.486 (22.074, 22.897) | 24.247 (24.035, 24.460) | <0.001 | <0.001 |

^a^ P-values for comparisons between cases and controls at each specific time point.

^b^ P-values adjusted using the False Discovery Rate (FDR) procedure.

**Appendix 8. Estimated marginal means of gait speed by case-control status (n=5,460)**

|  | **Estimated marginal means (95% CIs)** | |  |  |
| --- | --- | --- | --- | --- |
| **Time** | **Cases** | **Controls** | **P-contrast ^a^** | **Adjusted p-contrast ^b^** |
| **-11** | 1.068 (1.031, 1.105) | 1.117 (1.098, 1.135) | 0.02 | 0.03 |
| **-10** | 1.063 (1.034, 1.092) | 1.100 (1.085, 1.115) | 0.03 | 0.03 |
| **-9** | 1.054 (1.031, 1.077) | 1.083 (1.071, 1.094) | 0.03 | 0.03 |
| **-8** | 1.040 (1.022, 1.058) | 1.065 (1.055, 1.074) | 0.02 | 0.02 |
| **-7** | 1.022 (1.008, 1.037) | 1.046 (1.038, 1.053) | 0.005 | 0.007 |
| **-6** | 1.000 (0.987, 1.013) | 1.026 (1.020, 1.033) | <0.001 | <0.001 |
| **-5** | 0.973 (0.961, 0.985) | 1.006 (1.000, 1.012) | <0.001 | <0.001 |
| **-4** | 0.942 (0.930, 0.954) | 0.985 (0.979, 0.991) | <0.001 | <0.001 |
| **-3** | 0.906 (0.895, 0.918) | 0.964 (0.958, 0.970) | <0.001 | <0.001 |
| **-2** | 0.866 (0.855, 0.877) | 0.942 (0.936, 0.947) | <0.001 | <0.001 |
| **-1** | 0.822 (0.810, 0.834) | 0.919 (0.913, 0.925) | <0.001 | <0.001 |
| **0** | 0.773 (0.760, 0.787) | 0.895 (0.888, 0.902) | <0.001 | <0.001 |

^a^ P-values for comparisons between cases and controls at each specific time point.

^b^ P-values adjusted using the False Discovery Rate (FDR) procedure.

**Appendix 9. Estimated marginal means of frailty by case-control status in males (n=2,495)**

|  | **Estimated marginal means (95% CIs)** | |  |  |
| --- | --- | --- | --- | --- |
| **Time** | **Cases** | **Controls** | **P-contrast ^a^** | **Adjusted p-contrast ^b^** |
| **-11** | 0.105 (0.090, 0.120) | 0.091 (0.083, 0.098) | 0.06 | 0.21 |
| **-10** | 0.098 (0.086, 0.109) | 0.089 (0.083, 0.095) | 0.19 | 0.38 |
| **-9** | 0.093 (0.084, 0.102) | 0.088 (0.084, 0.093) | 0.40 | 0.60 |
| **-8** | 0.090 (0.083, 0.097) | 0.089 (0.085, 0.092) | 0.73 | 0.94 |
| **-7** | 0.090 (0.084, 0.095) | 0.090 (0.087, 0.093) | 0.94 | 0.94 |
| **-6** | 0.092 (0.087, 0.097) | 0.092 (0.090, 0.095) | 0.88 | 0.94 |
| **-5** | 0.097 (0.091, 0.102) | 0.096 (0.093, 0.098) | 0.81 | 0.94 |
| **-4** | 0.104 (0.098, 0.109) | 0.100 (0.098, 0.103) | 0.28 | 0.48 |
| **-3** | 0.113 (0.108, 0.119) | 0.106 (0.103, 0.109) | 0.02 | 0.07 |
| **-2** | 0.125 (0.119, 0.131) | 0.113 (0.110, 0.116) | <0.001 | <0.001 |
| **-1** | 0.139 (0.133, 0.146) | 0.120 (0.117, 0.124) | <0.001 | <0.001 |
| **0** | 0.156 (0.149, 0.163) | 0.129 (0.125, 0.133) | <0.001 | <0.001 |

^a^ P-values for comparisons between cases and controls at each specific time point.

^b^ P-values adjusted using the False Discovery Rate (FDR) procedure.

**Appendix 10. Estimated marginal means of frailty by case-control status in females (n=2,965)**

|  | **Estimated marginal means (95% CIs)** | |  |  |
| --- | --- | --- | --- | --- |
| **Time** | **Cases** | **Controls** | **P-contrast ^a^** | **Adjusted p-contrast ^b^** |
| **-11** | 0.123 (0.108, 0.138) | 0.096 (0.089, 0.104) | 0.001 | 0.002 |
| **-10** | 0.121 (0.109, 0.132) | 0.100 (0.094, 0.106) | 0.002 | 0.002 |
| **-9** | 0.120 (0.111, 0.129) | 0.104 (0.099, 0.108) | 0.002 | 0.002 |
| **-8** | 0.121 (0.114, 0.128) | 0.108 (0.104, 0.111) | 0.001 | 0.002 |
| **-7** | 0.123 (0.117, 0.129) | 0.112 (0.109, 0.116) | 0.002 | 0.002 |
| **-6** | 0.128 (0.122, 0.133) | 0.117 (0.115, 0.120) | 0.002 | 0.002 |
| **-5** | 0.133 (0.128, 0.139) | 0.123 (0.120, 0.126) | <0.001 | 0.002 |
| **-4** | 0.141 (0.135, 0.147) | 0.129 (0.126, 0.132) | <0.001 | <0.001 |
| **-3** | 0.150 (0.144, 0.156) | 0.135 (0.132, 0.139) | <0.001 | <0.001 |
| **-2** | 0.161 (0.155, 0.167) | 0.142 (0.139, 0.146) | <0.001 | <0.001 |
| **-1** | 0.174 (0.167, 0.180) | 0.150 (0.146, 0.153) | <0.001 | <0.001 |
| **0** | 0.188 (0.180, 0.195) | 0.157 (0.154, 0.161) | <0.001 | <0.001 |

^a^ P-values for comparisons between cases and controls at each specific time point.

^b^ P-values adjusted using the False Discovery Rate (FDR) procedure.

**Appendix 11. Estimated marginal means of grip strength by case-control status in males (n=2,495)**

|  | **Estimated marginal means (95% CIs)** | |  |  |
| --- | --- | --- | --- | --- |
| **Time** | **Cases** | **Controls** | **P-contrast ^a^** | **Adjusted p-contrast ^b^** |
| **-11** | 37.046 (35.456, 38.636) | 37.501 (36.708, 38.294) | 0.62 | 0.62 |
| **-10** | 36.518 (35.244, 37.792) | 36.874 (36.236, 37.512) | 0.62 | 0.62 |
| **-9** | 35.934 (34.907, 36.961) | 36.239 (35.723, 36.756) | 0.60 | 0.62 |
| **-8** | 35.295 (34.443, 36.146) | 35.597 (35.166, 36.028) | 0.53 | 0.62 |
| **-7** | 34.599 (33.857, 35.342) | 34.947 (34.569, 35.325) | 0.41 | 0.61 |
| **-6** | 33.849 (33.161, 34.536) | 34.289 (33.938, 34.640) | 0.26 | 0.44 |
| **-5** | 33.042 (32.380, 33.705) | 33.623 (33.284, 33.962) | 0.12 | 0.24 |
| **-4** | 32.180 (31.531, 32.829) | 32.949 (32.616, 33.281) | 0.04 | 0.09 |
| **-3** | 31.263 (30.624, 31.901) | 32.267 (31.940, 32.595) | 0.005 | 0.02 |
| **-2** | 30.289 (29.656, 30.922) | 31.578 (31.252, 31.903) | <0.001 | <0.001 |
| **-1** | 29.260 (28.611, 29.910) | 30.880 (30.547, 31.214) | <0.001 | <0.001 |
| **0** | 28.176 (27.461, 28.890) | 30.175 (29.810, 30.539) | <0.001 | <0.001 |

^a^ P-values for comparisons between cases and controls at each specific time point.

^b^ P-values adjusted using the False Discovery Rate (FDR) procedure.

**Appendix 12. Estimated marginal means of grip strength by case-control status in females (n=2,965)**

|  | **Estimated marginal means (95% CIs)** | |  |  |
| --- | --- | --- | --- | --- |
| **Time** | **Cases** | **Controls** | **P-contrast ^a^** | **Adjusted p-contrast ^b^** |
| **-11** | 21.285 (20.163, 22.406) | 21.408 (20.841, 21.974) | 0.85 | 0.85 |
| **-10** | 20.987 (20.095, 21.879) | 21.246 (20.795, 21.697) | 0.61 | 0.67 |
| **-9** | 20.664 (19.954, 21.373) | 21.059 (20.700, 21.418) | 0.33 | 0.39 |
| **-8** | 20.315 (19.740, 20.891) | 20.846 (20.554, 21.139) | 0.10 | 0.14 |
| **-7** | 19.942 (19.453, 20.431) | 20.608 (20.358, 20.858) | 0.02 | 0.02 |
| **-6** | 19.543 (19.102, 19.984) | 20.344 (20.118, 20.571) | 0.001 | 0.002 |
| **-5** | 19.119 (18.701, 19.537) | 20.055 (19.840, 20.271) | <0.001 | <0.001 |
| **-4** | 18.670 (18.266, 19.074) | 19.741 (19.532, 19.949) | <0.001 | <0.001 |
| **-3** | 18.196 (17.803, 18.588) | 19.401 (19.197, 19.604) | <0.001 | <0.001 |
| **-2** | 17.696 (17.311, 18.081) | 19.035 (18.835, 19.235) | <0.001 | <0.001 |
| **-1** | 17.172 (16.775, 17.568) | 18.644 (18.438, 18.850) | <0.001 | <0.001 |
| **0** | 16.622 (16.176, 17.067) | 18.227 (17.996, 18.459) | <0.001 | <0.001 |

^a^ P-values for comparisons between cases and controls at each specific time point.

^b^ P-values adjusted using the False Discovery Rate (FDR) procedure.

**Appendix 13. Estimated marginal means of gait speed by case-control status in males (n=2,495)**

|  | **Estimated marginal means (95% CIs)** | |  |  |
| --- | --- | --- | --- | --- |
| **Time** | **Cases** | **Controls** | **P-contrast ^a^** | **Adjusted p-contrast ^b^** |
| **-11** | 1.088 (1.032, 1.143) | 1.138 (1.110, 1.166) | 0.11 | 0.17 |
| **-10** | 1.088 (1.044, 1.132) | 1.124 (1.102, 1.146) | 0.15 | 0.20 |
| **-9** | 1.084 (1.050, 1.118) | 1.109 (1.092, 1.126) | 0.19 | 0.21 |
| **-8** | 1.075 (1.048, 1.102) | 1.093 (1.080, 1.107) | 0.22 | 0.22 |
| **-7** | 1.060 (1.038, 1.082) | 1.077 (1.066, 1.088) | 0.19 | 0.21 |
| **-6** | 1.041 (1.022, 1.060) | 1.059 (1.049, 1.069) | 0.10 | 0.17 |
| **-5** | 1.017 (0.999, 1.035) | 1.041 (1.032, 1.050) | 0.02 | 0.04 |
| **-4** | 0.988 (0.971, 1.005) | 1.021 (1.013, 1.030) | <0.001 | <0.001 |
| **-3** | 0.953 (0.937, 0.970) | 1.001 (0.993, 1.009) | <0.001 | <0.001 |
| **-2** | 0.914 (0.898, 0.930) | 0.980 (0.971, 0.988) | <0.001 | <0.001 |
| **-1** | 0.870 (0.853, 0.886) | 0.958 (0.949, 0.966) | <0.001 | <0.001 |
| **0** | 0.820 (0.800, 0.840) | 0.935 (0.925, 0.945) | <0.001 | <0.001 |

^a^ P-values for comparisons between cases and controls at each specific time point.

^b^ P-values adjusted using the False Discovery Rate (FDR) procedure.

**Appendix 14. Estimated marginal means of gait speed by case-control status in females (n=2,965)**

|  | **Estimated marginal means (95% CIs)** | |  |  |
| --- | --- | --- | --- | --- |
| **Time** | **Cases** | **Controls** | **P-contrast ^a^** | **Adjusted p-contrast ^b^** |
| **-11** | 1.047 (0.997, 1.097) | 1.092 (1.066, 1.117) | 0.12 | 0.12 |
| **-10** | 1.037 (0.998, 1.076) | 1.073 (1.053, 1.093) | 0.11 | 0.12 |
| **-9** | 1.023 (0.993, 1.054) | 1.054 (1.038, 1.069) | 0.08 | 0.10 |
| **-8** | 1.006 (0.982, 1.030) | 1.034 (1.022, 1.046) | 0.04 | 0.06 |
| **-7** | 0.985 (0.965, 1.005) | 1.014 (1.003, 1.024) | 0.01 | 0.02 |
| **-6** | 0.959 (0.942, 0.977) | 0.993 (0.984, 1.002) | <0.001 | 0.002 |
| **-5** | 0.931 (0.914, 0.947) | 0.971 (0.963, 0.980) | <0.001 | <0.001 |
| **-4** | 0.898 (0.882, 0.914) | 0.949 (0.941, 0.958) | <0.001 | <0.001 |
| **-3** | 0.861 (0.845, 0.877) | 0.927 (0.919, 0.935) | <0.001 | <0.001 |
| **-2** | 0.821 (0.805, 0.836) | 0.904 (0.896, 0.912) | <0.001 | <0.001 |
| **-1** | 0.776 (0.760, 0.792) | 0.880 (0.872, 0.889) | <0.001 | <0.001 |
| **0** | 0.728 (0.709, 0.747) | 0.856 (0.846, 0.866) | <0.001 | <0.001 |

^a^ P-values for comparisons between cases and controls at each specific time point.

^b^ P-values adjusted using the False Discovery Rate (FDR) procedure.

**Appendix 15. Estimated marginal means of frailty by case-control status in ApoE ε4 carriers (n=1,197)**

|  | **Estimated marginal means (95% CIs)** | |  |  |
| --- | --- | --- | --- | --- |
| **Time** | **Cases** | **Controls** | **P-contrast ^a^** | **Adjusted p-contrast ^b^** |
| **-11** | 0.093 (0.076, 0.110) | 0.092 (0.081, 0.102) | 0.89 | 0.89 |
| **-10** | 0.093 (0.079, 0.106) | 0.091 (0.083, 0.100) | 0.88 | 0.89 |
| **-9** | 0.094 (0.083, 0.104) | 0.092 (0.085, 0.099) | 0.82 | 0.89 |
| **-8** | 0.096 (0.087, 0.104) | 0.094 (0.088, 0.099) | 0.69 | 0.89 |
| **-7** | 0.099 (0.092, 0.106) | 0.096 (0.091, 0.100) | 0.48 | 0.72 |
| **-6** | 0.103 (0.097, 0.110) | 0.099 (0.095, 0.103) | 0.26 | 0.44 |
| **-5** | 0.109 (0.103, 0.115) | 0.103 (0.099, 0.107) | 0.11 | 0.22 |
| **-4** | 0.116 (0.109, 0.122) | 0.108 (0.103, 0.112) | 0.04 | 0.08 |
| **-3** | 0.123 (0.117, 0.130) | 0.113 (0.109, 0.117) | 0.009 | 0.03 |
| **-2** | 0.133 (0.126, 0.140) | 0.119 (0.115, 0.124) | 0.002 | 0.008 |
| **-1** | 0.143 (0.135, 0.150) | 0.126 (0.121, 0.131) | <0.001 | <0.001 |
| **0** | 0.154 (0.146, 0.163) | 0.134 (0.128, 0.140) | <0.001 | <0.001 |

^a^ P-values for comparisons between cases and controls at each specific time point.

^b^ P-values adjusted using the False Discovery Rate (FDR) procedure.

**Appendix 16. Estimated marginal means of frailty by case-control status in ApoE ε4 non-carriers (n=3,151)**

|  | **Estimated marginal means (95% CIs)** | |  |  |
| --- | --- | --- | --- | --- |
| **Time** | **Cases** | **Controls** | **P-contrast ^a^** | **Adjusted p-contrast ^b^** |
| **-11** | 0.124 (0.109, 0.139) | 0.092 (0.086, 0.099) | <0.001 | <0.001 |
| **-10** | 0.115 (0.104, 0.127) | 0.093 (0.088, 0.098) | <0.001 | 0.002 |
| **-9** | 0.110 (0.101, 0.119) | 0.095 (0.091, 0.099) | 0.003 | 0.005 |
| **-8** | 0.107 (0.100, 0.115) | 0.097 (0.094, 0.100) | 0.02 | 0.02 |
| **-7** | 0.107 (0.101, 0.113) | 0.100 (0.098, 0.103) | 0.05 | 0.05 |
| **-6** | 0.110 (0.104, 0.116) | 0.104 (0.102, 0.107) | 0.08 | 0.08 |
| **-5** | 0.115 (0.110, 0.121) | 0.109 (0.106, 0.111) | 0.05 | 0.05 |
| **-4** | 0.124 (0.118, 0.130) | 0.114 (0.112, 0.117) | 0.006 | 0.009 |
| **-3** | 0.135 (0.128, 0.141) | 0.121 (0.118, 0.123) | <0.001 | <0.001 |
| **-2** | 0.148 (0.142, 0.155) | 0.128 (0.125, 0.131) | <0.001 | <0.001 |
| **-1** | 0.165 (0.157, 0.172) | 0.135 (0.132, 0.139) | <0.001 | <0.001 |
| **0** | 0.184 (0.176, 0.192) | 0.144 (0.140, 0.147) | <0.001 | <0.001 |

^a^ P-values for comparisons between cases and controls at each specific time point.

^b^ P-values adjusted using the False Discovery Rate (FDR) procedure.

**Appendix 17. Estimated marginal means of grip strength by case-control status in ApoE ε4 carriers (n=1,197)**

|  | **Estimated marginal means (95% CIs)** | |  |  |
| --- | --- | --- | --- | --- |
| **Time** | **Cases** | **Cases** | **P-contrast ^a^** | **Adjusted p-contrast ^b^** |
| **-11** | 30.734 (29.013, 32.455) | 30.476 (29.390, 31.563) | 0.80 | 0.88 |
| **-10** | 29.968 (28.595, 31.342) | 29.957 (29.085, 30.829) | 0.96 | 0.99 |
| **-9** | 29.210 (28.111, 30.309) | 29.435 (28.730, 30.139) | 0.73 | 0.88 |
| **-8** | 28.459 (27.560, 29.357) | 28.909 (28.325, 29.494) | 0.41 | 0.54 |
| **-7** | 27.714 (26.945, 28.484) | 28.381 (27.873, 28.890) | 0.15 | 0.22 |
| **-6** | 26.977 (26.277, 27.677) | 27.851 (27.383, 28.318) | 0.04 | 0.07 |
| **-5** | 26.247 (25.582, 26.913) | 27.317 (26.871, 27.764) | 0.008 | 0.02 |
| **-4** | 25.524 (24.879, 26.170) | 26.781 (26.348, 27.214) | 0.001 | 0.002 |
| **-3** | 24.809 (24.180, 25.437) | 26.242 (25.820, 26.663) | <0.001 | <0.001 |
| **-2** | 24.100 (23.483, 24.717) | 25.700 (25.285, 26.114) | <0.001 | <0.001 |
| **-1** | 23.399 (22.768, 24.029) | 25.155 (24.731, 25.579) | <0.001 | <0.001 |
| **0** | 22.704 (22.005, 23.403) | 24.607 (24.137, 25.077) | <0.001 | <0.001 |

^a^ P-values for comparisons between cases and controls at each specific time point.

^b^ P-values adjusted using the False Discovery Rate (FDR) procedure.

**Appendix 18. Estimated marginal means of grip strength by case-control status in ApoE ε4 non-carriers (n=3,151)**

|  | **Estimated marginal means (95% CIs)** | |  |  |
| --- | --- | --- | --- | --- |
| **Time** | **Cases** | **Controls** | **P-contrast ^a^** | **Adjusted p-contrast ^b^** |
| **-11** | 28.065 (26.721, 29.410) | 29.204 (28.603, 29.805) | 0.13 | 0.13 |
| **-10** | 27.940 (26.863, 29.018) | 28.877 (28.396, 29.358) | 0.12 | 0.13 |
| **-9** | 27.738 (26.870, 28.607) | 28.524 (28.138, 28.911) | 0.10 | 0.13 |
| **-8** | 27.459 (26.739, 28.179) | 28.148 (27.829, 28.466) | 0.09 | 0.11 |
| **-7** | 27.103 (26.474, 27.731) | 27.746 (27.470, 28.022) | 0.06 | 0.10 |
| **-6** | 26.669 (26.087, 27.251) | 27.320 (27.066, 27.574) | 0.04 | 0.07 |
| **-5** | 26.158 (25.595, 26.721) | 26.869 (26.625, 27.114) | 0.02 | 0.04 |
| **-4** | 25.570 (25.016, 26.124) | 26.394 (26.154, 26.634) | 0.007 | 0.02 |
| **-3** | 24.905 (24.357, 25.453) | 25.894 (25.657, 26.132) | 0.001 | 0.003 |
| **-2** | 24.162 (23.614, 24.711) | 25.370 (25.133, 25.607) | <0.001 | <0.001 |
| **-1** | 23.343 (22.773, 23.912) | 24.821 (24.575, 25.067) | <0.001 | <0.001 |
| **0** | 22.446 (21.814, 23.077) | 24.247 (23.975, 24.519) | <0.001 | <0.001 |

^a^ P-values for comparisons between cases and controls at each specific time point.

^b^ P-values adjusted using the False Discovery Rate (FDR) procedure.

**Appendix 19. Estimated marginal means of gait speed by case-control status in ApoE ε4 carriers (n=1,197)**

|  | **Estimated marginal means (95% CIs)** | |  |  |
| --- | --- | --- | --- | --- |
| **Time** | **Cases** | **Controls** | **P-contrast ^a^** | **Adjusted p-contrast ^b^** |
| **-11** | 1.098 (1.031, 1.165) | 1.134 (1.092, 1.176) | 0.37 | 0.59 |
| **-10** | 1.093 (1.040, 1.146) | 1.116 (1.083, 1.149) | 0.46 | 0.62 |
| **-9** | 1.083 (1.042, 1.124) | 1.098 (1.072, 1.124) | 0.56 | 0.62 |
| **-8** | 1.070 (1.038, 1.102) | 1.079 (1.059, 1.100) | 0.62 | 0.62 |
| **-7** | 1.052 (1.025, 1.078) | 1.060 (1.043, 1.078) | 0.58 | 0.62 |
| **-6** | 1.029 (1.006, 1.052) | 1.041 (1.026, 1.056) | 0.40 | 0.59 |
| **-5** | 1.003 (0.981, 1.024) | 1.021 (1.007, 1.036) | 0.15 | 0.29 |
| **-4** | 0.971 (0.951, 0.992) | 1.001 (0.987, 1.015) | 0.02 | 0.04 |
| **-3** | 0.936 (0.916, 0.956) | 0.980 (0.967, 0.994) | <0.001 | <0.001 |
| **-2** | 0.896 (0.877, 0.915) | 0.959 (0.947, 0.972) | <0.001 | <0.001 |
| **-1** | 0.852 (0.832, 0.872) | 0.938 (0.925, 0.951) | <0.001 | <0.001 |
| **0** | 0.804 (0.780, 0.827) | 0.916 (0.900, 0.932) | <0.001 | <0.001 |

^a^ P-values for comparisons between cases and controls at each specific time point.

^b^ P-values adjusted using the False Discovery Rate (FDR) procedure.

**Appendix 20. Estimated marginal means of gait speed by case-control status in ApoE ε4 non-carriers (n=3,151)**

|  | **Estimated marginal means (95% CIs)** | |  |  |
| --- | --- | --- | --- | --- |
| **Time** | **Cases** | **Controls** | **P-contrast ^a^** | **Adjusted p-contrast ^b^** |
| **-11** | 1.075 (1.023, 1.128) | 1.119 (1.096, 1.143) | 0.13 | 0.13 |
| **-10** | 1.066 (1.025, 1.107) | 1.103 (1.084, 1.121) | 0.11 | 0.12 |
| **-9** | 1.053 (1.021, 1.085) | 1.086 (1.071, 1.100) | 0.07 | 0.09 |
| **-8** | 1.036 (1.011, 1.062) | 1.068 (1.056, 1.079) | 0.03 | 0.04 |
| **-7** | 1.016 (0.995, 1.037) | 1.049 (1.039, 1.058) | 0.005 | 0.007 |
| **-6** | 0.992 (0.973, 1.011) | 1.029 (1.021, 1.038) | <0.001 | <0.001 |
| **-5** | 0.963 (0.946, 0.981) | 1.009 (1.001, 1.017) | <0.001 | <0.001 |
| **-4** | 0.932 (0.914, 0.949) | 0.988 (0.981, 0.996) | <0.001 | <0.001 |
| **-3** | 0.896 (0.879, 0.913) | 0.966 (0.959, 0.974) | <0.001 | <0.001 |
| **-2** | 0.856 (0.840, 0.873) | 0.944 (0.937, 0.951) | <0.001 | <0.001 |
| **-1** | 0.813 (0.795, 0.830) | 0.921 (0.913, 0.928) | <0.001 | <0.001 |
| **0** | 0.766 (0.745, 0.786) | 0.897 (0.888, 0.906) | <0.001 | <0.001 |

^a^ P-values for comparisons between cases and controls at each specific time point.

^b^ P-values adjusted using the False Discovery Rate (FDR) procedure.

**
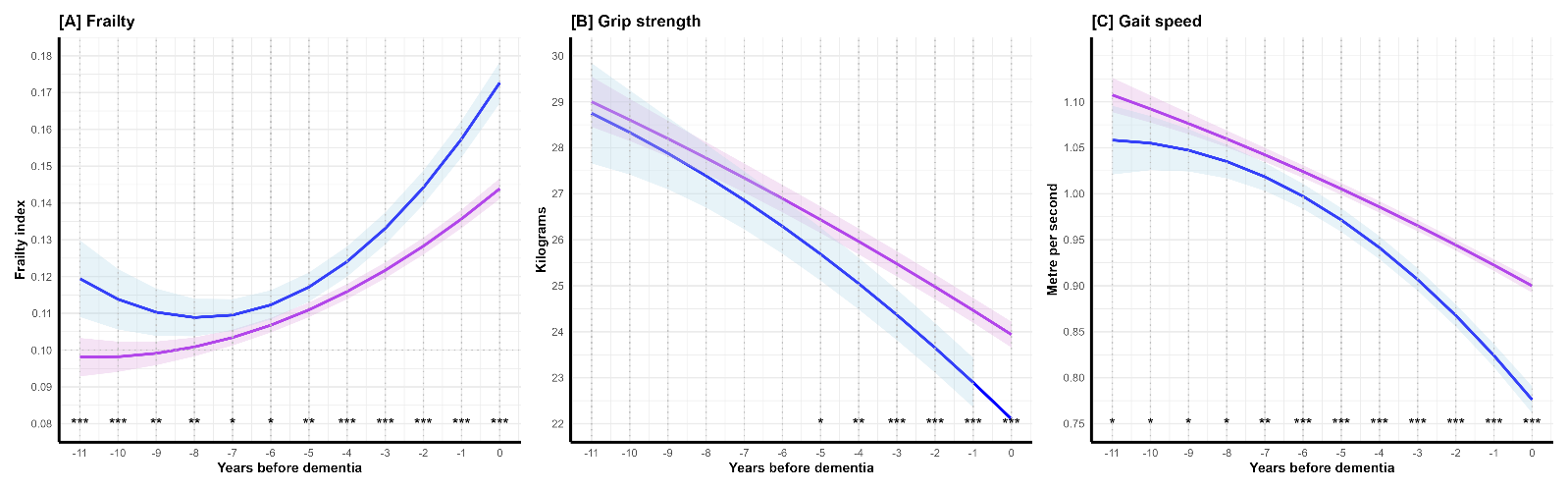
Appendix 21. Unadjusted mean trajectories of [A] frailty, [B] grip strength and [C] gait speed in cases preceding dementia and in matched controls (n=5,460)**

Note: 1) The solid lines and shadings represent the estimated mean trajectories and the 95% confidence intervals. 2) Cases are shown in blue and controls are shown in purple.

* P-contrast<0.05; ** P-contrast<0.01; *** P-contrast<0.001

**
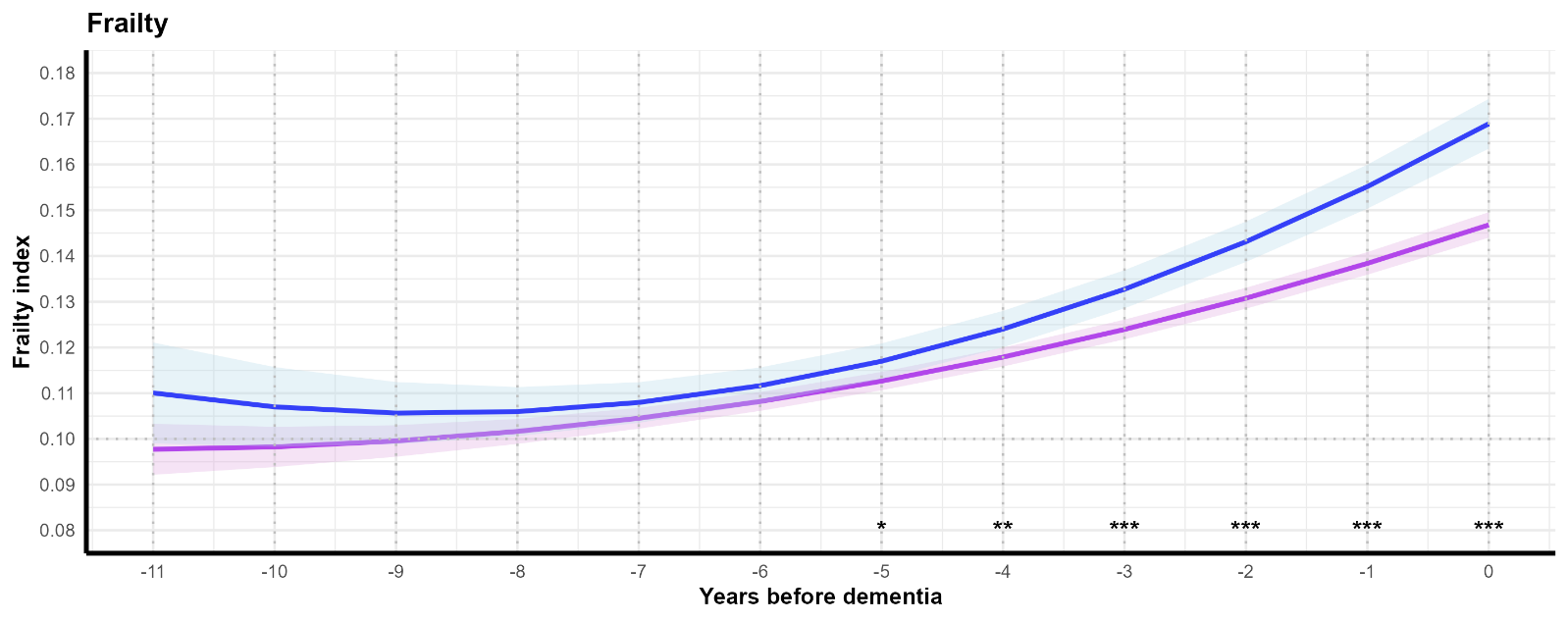
Appendix 22. Mean trajectories of frailty in cases preceding dementia and in matched controls, with cognitive components removed from the frailty index (n=5,875)**

Note: 1) The solid lines and shadings represent the estimated mean trajectories and the 95% confidence intervals. 2) Cases are shown in blue and controls are shown in purple. 3) Analysis was conducted in a re-matched case-control sample (n=5,875, 1,175 cases and 4,700 controls) drawn from the ASPREE participants.

* P-contrast<0.05; ** P-contrast<0.01; *** P-contrast<0.001

**
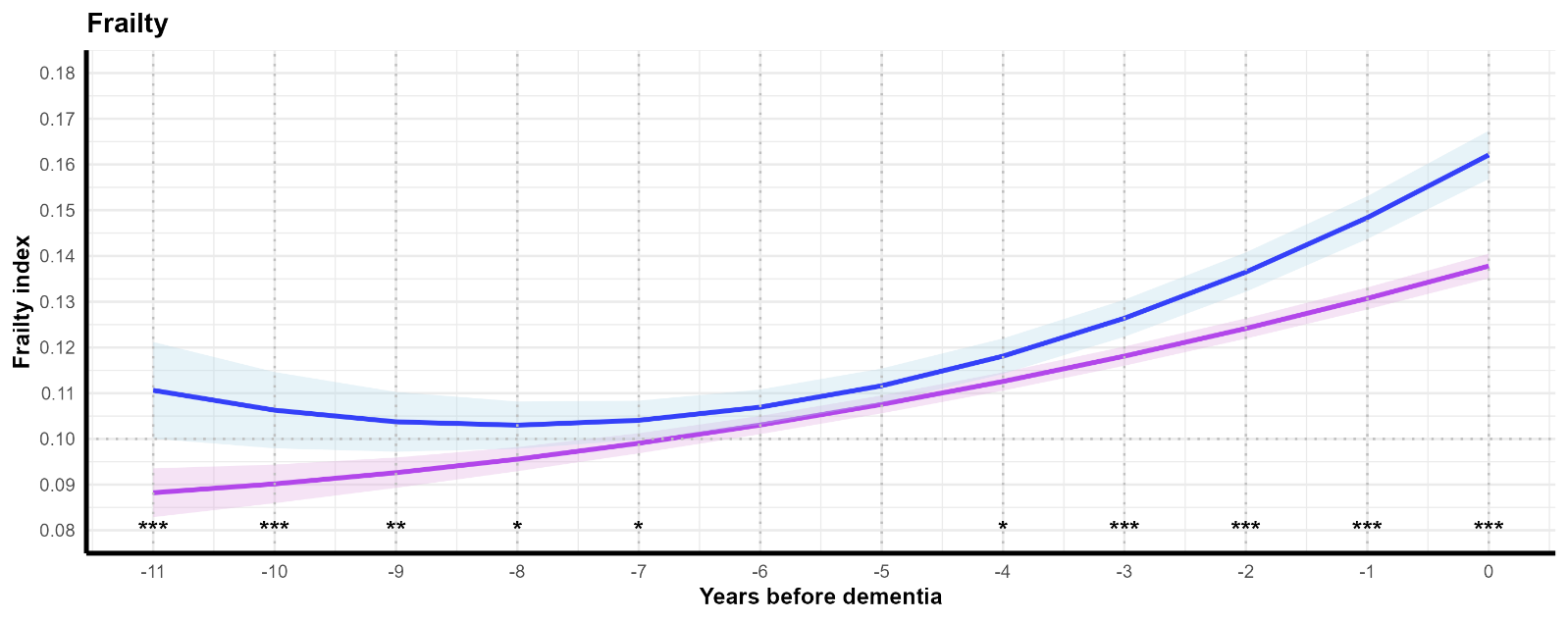
Appendix 23. Mean trajectories of frailty in cases preceding dementia and in matched controls, with grip strength and gait speed removed from the frailty index (n=5,895)**

Note: 1) The solid lines and shadings represent the estimated mean trajectories and the 95% confidence intervals. 2) Cases are shown in blue and controls are shown in purple. 3) Analysis was conducted in a re-matched case-control sample (n=5,895, 1,179 cases and 4,716 controls) drawn from the ASPREE participants.

* P-contrast<0.05; ** P-contrast<0.01; *** P-contrast<0.001

**
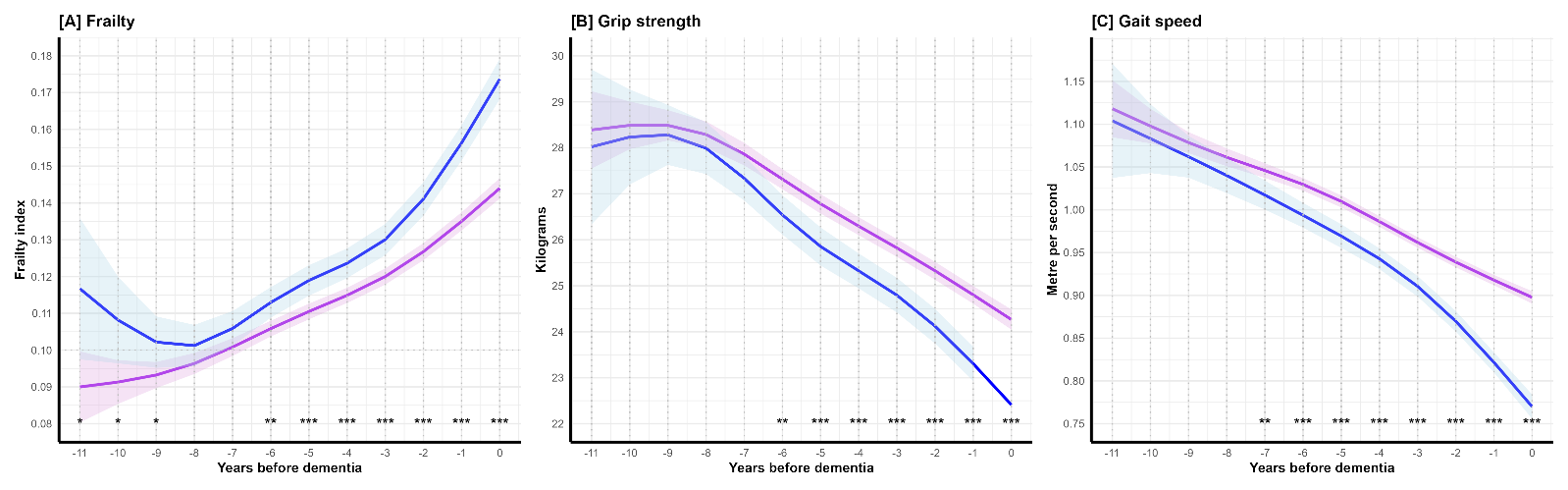
Appendix 24. Mean trajectories of [A] frailty, [B] grip strength and [C] gait speed in cases preceding dementia and in matched controls, using spline-based models (n=5,460)**

Note: 1) The solid lines and shadings represent the estimated mean trajectories and the 95% confidence intervals. 2) Cases are shown in blue and controls are shown in purple. 3) Models were fitted using natural cubic splines.

* P-contrast<0.05; ** P-contrast<0.01; *** P-contrast<0.001

**
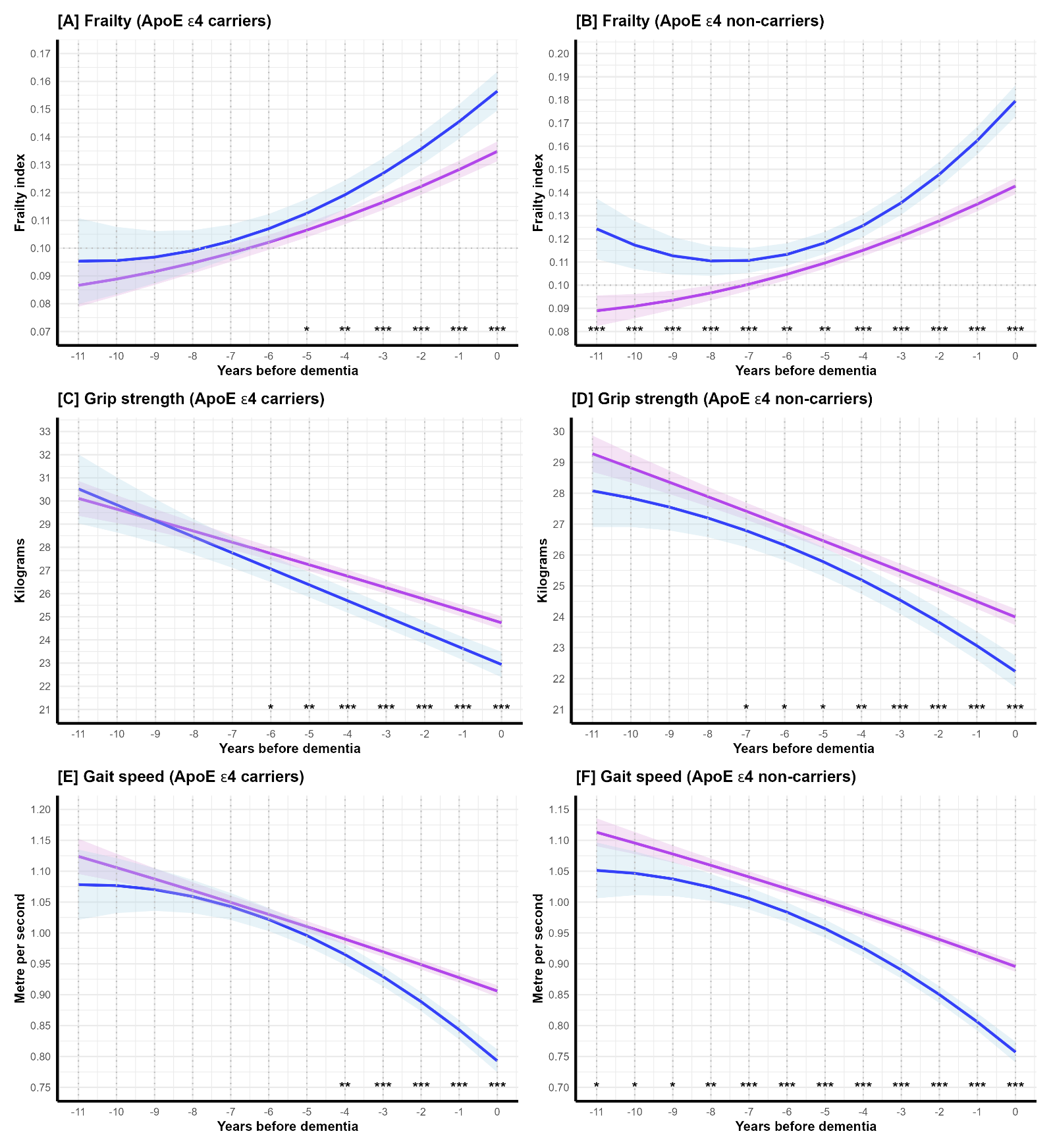
Appendix 25. Mean trajectories of [A][B] frailty, [C][D] grip strength and [E][F] gait speed preceding dementia and in matched controls, among ApoE ε4 carriers (n=3,035) and non-carriers (n=3,635)**

Note: 1) The solid lines and shadings represent the estimated mean trajectories and the 95% confidence intervals. 2) Cases are shown in blue and controls are shown in purple. 3) Analysis was conducted in two case-control samples, separately matched by ApoE ε4 carrier status, resulting in a sample of 3,035 ApoE ε4 carriers (607 cases and 2,428 controls) and 3,635 ApoE ε4 non-carriers (727 cases and 2,908 controls).

* P-contrast<0.05; ** P-contrast<0.01; *** P-contrast<0.001
